# Temperature Detectable Surface Coating with Carbon Nanotube/Epoxy Composites

**DOI:** 10.3390/nano12142369

**Published:** 2022-07-11

**Authors:** Seung-Jun Lee, Yu-Jin Jung, JeeWoong Park, Sung-Hwan Jang

**Affiliations:** 1 Department of Civil and Environmental Engineering, Hanyang University, Seoul 04763, Korea; sj5523@hanyang.ac.kr; 2 Department of Smart City Engineering, Hanyang University ERICA, Ansan 15588, Korea; yujin0421@hanyang.ac.kr; 3 Department of Civil and Environmental Engineering and Construction, The University of Nevada, Las Vegas, NV 89154, USA; 4 Department of Civil and Environmental Engineering, Hanyang University ERICA, Ansan 15588, Korea

**Keywords:** carbon nanotube, surface coating, temperature sensing, epoxy

## Abstract

In the construction and machinery industry, heat is a major factor causing damage and destruction. The safety and efficiency of most machines and structures are greatly affected by temperature, and temperature management and control are essential. In this study, a carbon nanotube (CNT) based temperature sensing coating that can be applied to machines and structures having various structural types was fabricated, and characteristics analysis and temperature sensing performance were evaluated. The surface coating, which detects temperature through resistance change is made of a nanocomposite composed of carbon nanotubes (CNT) and epoxy (EP). We investigated the electrical properties by CNT concentration and temperature sensing performance of CNT/EP coating against static and cyclic temperatures. In addition, the applicability of the CNT/EP coating was investigated through a partially heating and cooling experiment. As a result of the experiment, the CNT/EP coating showed higher electrical conductivity as the CNT concentration increased. In addition, the CNT/EP coating exhibits high sensing performance in the high and sub−zero temperature ranges with a negative temperature coefficient of resistance. Therefore, the proposed CNT/EP coatings are promising for use as multi-functional coating materials for the detection of high and freezing temperatures.

## 1. Introduction

Temperature is a key parameter for most industries, and temperature sensors account for a large part of the overall sensor market [1]. In addition, according to industrial development, a temperature sensor is required with capable of responding to complex structures and various environments. However, in the case of conventional temperature sensor materials such as silicon diodes and platinum, heat loss occurs when there is not enough thermal energy, resulting in poor precision or slow response. It is also difficult to cope with large areas. In order to compensate for the shortcomings of the conventional temperature sensors, research on new materials or composites is being actively conducted.

Carbon nanotubes (CNT) are widely used as a functional filler, and carbon nanotube composites are utilized in various industrial fields. The structural morphology of CNT can be used as a composite mixed with different materials to impart electrical and thermal conductivity and/or mechanical properties [2,3,4]. In particular, the dispersion of these kinds of nanoparticles in polymeric systems allows to improve thermal stability, photooxidation resistance, and mechanical properties and simultaneously provides the means to make the resulting nanocomposite able to manifest functional properties [5,6].

The effect of temperature on CNT reinforced composites has been studied for different filler-resin composites. Gojny and Schulte [7] investigated the effect of multi-walled CNTs on the thermo-mechanical properties of multi-wall carbon nanotubes (MWCNTs)/epoxy composites and found that increasing concentrations of MWCNTs as well as functionalizing MWCNTs leads to an increase of the glass transition temperature with higher interfacial interaction between the CNT and the polymer matrix. For example, Jang and Yin [8,9] fabricated highly sensitive strain and fracture sensors by dispersing carbon nanotubes as well as ferromagnetic particles in polydimethylsiloxane (PDMS). In addition to sensing applications, other capabilities have been reported for a decade. Jang and Park [10] proposed carbon nanotube-reinforced composite materials for dual functions such as temperature sensing and de-icing. Yum and Jang [11] proposed multi-functional road coating materials with self-sensing consisting of CNTs and a polyurethane (PU) matrix, widely used materials for road marking, for future transportation systems.

Among the various polymer systems, commonly used epoxy resins belong to structurally suitable polymer systems [12,13,14]. These resins have excellent stiffness, strength, dimensional stability, chemical resistance, and durability, making them useful for a wide variety of industrial applications, particularly in the electronics, automotive, and aerospace industries [15,16]. Alamusi et al. [17] investigated the temperature dependence of CNT/epoxy resin composites resistance. Wenlong Wang et al. [18] investigated the correlation of the strain variation in CNT/epoxy resin composites with temperature using Raman spectroscopy.

In this study, a novel temperature-sensing coating composed of carbon nanotube and epoxy (EP) matrices applicable to various industrial fields was proposed. The CNT/EP coating material is manufactured by the solution casting method for high electrical conductivity. We then investigated the electrical conductivity of the CNT/EP coatings as a function of CNT concentration. The temperature sensing accuracy, sensing speed, and repeatability of the proposed CNT/EP coating were investigated through resistance measurement experiments for static and cyclic temperatures. In addition, applicability was evaluated by measuring the response of the CNT/EP coating to freezing and high temperature.

## 2. Experimental

### 2.1. Materials

Industrial-grade multi-walled carbon nanotubes were purchased from Nanolab, Inc. (Waltham, MA, USA). The CNTs have a diameter of 15 ± 5 nm, a length of 5–20 µm, and purity of higher than 85 wt.% including impurities such as iron and ceramic oxides. Epoxy was obtained from Easy Composites Ltd. (EpoxAcast 690, Staffordshire, UK) with a density of 1.12–1.18 g/cm^3^ and viscosity of 200–450 mPa∙s. The dispersant used acetone from Samchun pure chemical Co., Ltd. (Gyeonggi-do, Korea) with a purity of 99.7%.

### 2.2. Fabrication Procedure

**Figure 1:**
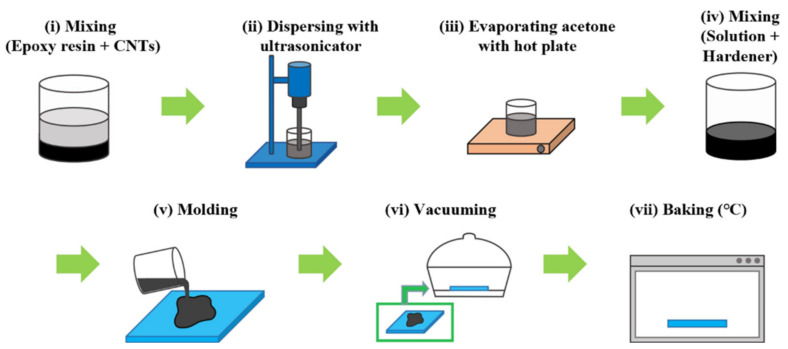
Fabrication of CNT/EP coating.

Various fabrication methods according to matrix materials are being studied for the carbon nanotube-based composites [19,20]. In this study, the CNT/EP coating was fabricated based on the previous study [8,9,10,20]. The fabrication procedure of CNT/EP coatings was shown in Figure 1. 20 g of epoxy resin and 50 g of acetone were added to a 200 mL beaker and hand-mixed using a spatula. Then, various concentrations of CNTs (0–5 wt.%) were put into a beaker and mixed in the same way. An ultrasonicator (Q700CA, Qsonica LLC., Newtown, CT, USA) was applied to disperse CNTs in the solution. In this study, the ultrasonicator was operated in a pulsed mode with 90% amplitude for 30 min. Ice was placed around the beaker to prevent the evaporation of acetone. After dispersion, the sample was placed on a hot plate at 60 °C for 24 h to fully evaporate acetone. Then, 6 g of curing agent was added into the sample and was mixed evenly in a 3-roll mill (TR 50M, Trilos, San Ramon, CA, USA). After molding, the sample was placed into the vacuum chamber and operated for 30 min to remove air bubbles inside the sample. Finally, the CNT/EP coating was baked in hot oven at 60 °C.

### 2.3. Characterization

The resistance of the CNT/EP coatings was measured using a Keithley 2450 (Tektronix, Beaverton, OR, USA) for a high resistance above 10^9^ Ω and Keithley 2700 (Tektronix, Beaverton, OR, USA) for normal resistance. The electrical resistance was evaluated from the current-voltage curves obtained by applying voltages between −10 V and +10 V. The specimens were prepared with a size of 50 mm × 30 mm × 1 mm. High-purity silver paint was applied to both ends of the specimens to minimize the contact resistance between the coating and the tip probe. The electrical conductivity (σ) of the specimens was calculated by

σ = L/RA(1)

where R is the resistance of the coating (Ω), A is the area of the coating (m^2^) and L is the length of the coating (m).

For the microstructure of the CNT/EP coatings, the cross-section of the specimen was observed through a scanning electron microscope (MIRA3 FE-SEMs, TESCAN, Brno, Czech Republic) at 15 kV. For a high magnification measurement of more than 10,000 times, the CNT/EP coating cross-section was coated with platinum using sputter coating (QUORUM−Q150T S, Laughton, UK) for 10 min.

**Figure 2:**
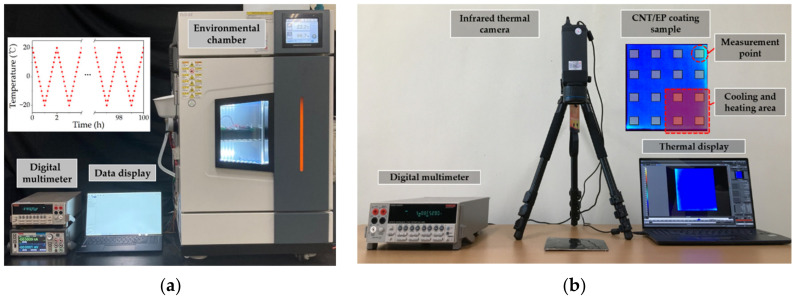
Temperature sensing performance equipment and details: (**a**) static and cycle sensing test; (**b**) multi-sensing test.

For resistance-temperature dependence in CNT/EP coatings, the change in resistance of CNT/EP coatings as a function of temperature was measured in an environmental chamber using a digital multimeter (Keithley 2700, Tektronix, Beaverton, OR, USA) with an externally connected data acquisition system, as shown in Figure 2a. The resistance of CNT/EP coatings was measured from −20 °C to 60 °C in 10 degree increments. Note that the samples remain constant at each temperature for 1 h for the steady-state temperature of the CNT/EP coatings. For cyclic piezoresistive response of CNT/EP coatings, the test exposed the samples repeatedly to sub-zero temperature ranges of −20 °C and +20 °C, where atmospheric phase changes can occur. The temperature change will cycle 50 times throughout the test with an hour dwell at each level.

For multi-sensing application, CNT/EP coating (5.0 wt.%) was painted on a polyethylene terephthalate (PET) substrate with a size of 150 mm × 150 mm × 10.0 mm. Total of 16 sensors were prepared using a silver paint and copper tape, as shown in Figure 2b. Heat source was applied to a side of PET substrate. The resistance of 16 sensors was collected using a digital multimeter.

## 3. Results and Discussion

### 3.1. Electrical Conductivity of CNT/EP Coating

**Figure 3:**
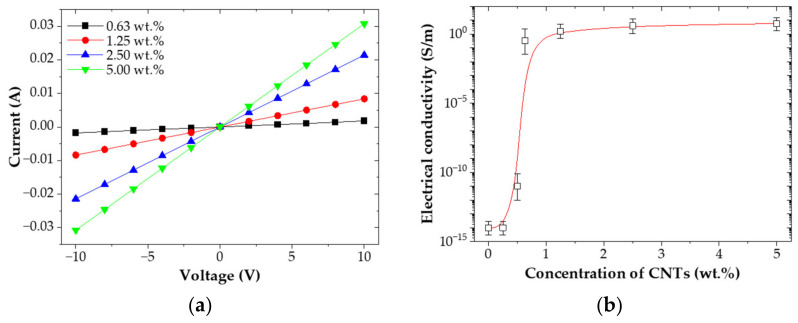

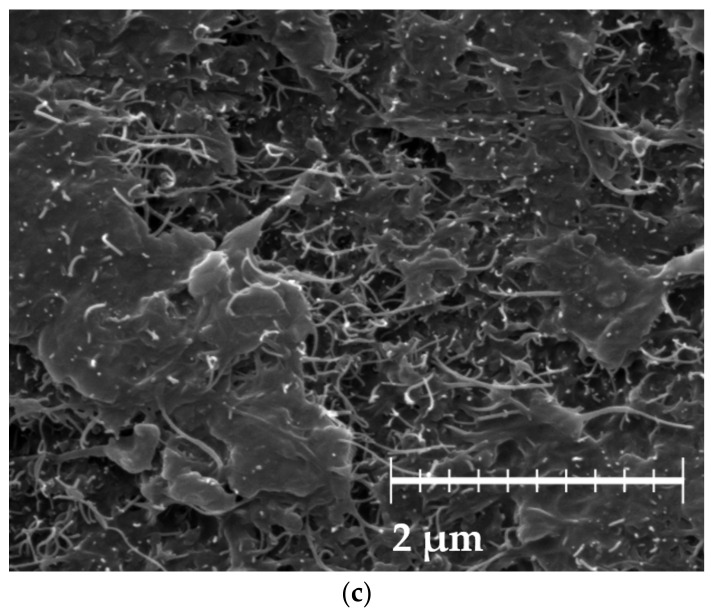
Electrical characteristics of CNT/EP coating; (**a**) current−voltage curves; (**b**) electrical conductivity; (**c**) SEM image.

Figure 3 showed the electrical characteristics of the CNT/EP coatings. Figure 3a showed the current–voltage curves. It can be seen that negative and positive voltage sections of the current–voltage curve are symmetrical and have linear relationships with each other. This indicates the good ohmic behavior of the CNT/EP coatings. Figure 3b showed the electrical conductivity for the CNT/EP coating as a function of CNT concentrations. Pure epoxy was non-conductive material with 1 × 10^−14^ S/m. A dramatic increase in electrical conductivity was observed when the concentration of CNTs increased from 0.25 wt.% to 1.0 wt.%. This behavior has been attributed to the occurrence of a percolation threshold [21,22,23,24]. In this study the percolation threshold, i.e., the minimum CNT concentration in the matrix after which no significant change in the electrical conductivity is observed, occurred at around 0.63 wt.% CNTs. Then, the electrical conductivity showed a slight increase even with an increase in the CNT concentrations. The observed increase in electrical conductivity of the CNT/EP coating is due to a well-dispersed CNT into the epoxy matrix as shown in Figure 3c. The effectiveness of electron transfer between the CNTs is very highly dependent on these CNTs’ spacing distance. CNT/EP Coating does not reach the improved percolation network formation of multi-wall carbon nanotubes but exhibits excellent electrical conductivity [25].

**Figure 4:**
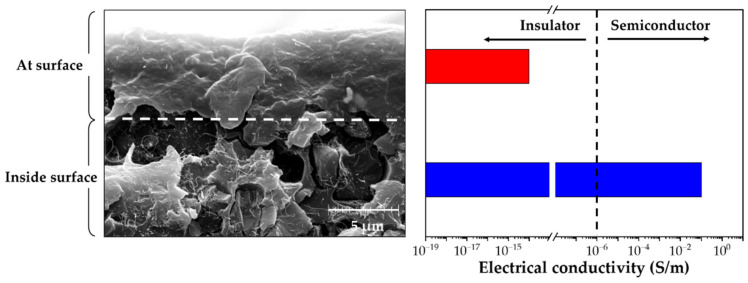
SEM image of a cross-section of the CNT/EP coating with electrical conductivity.

Figure 4 showed an SEM image of a cross-section of the CNT/EP coating with electrical conductivity. Densely packed CNT networks were clearly observed inside the sample. The electrical conductivity of the sample at the surface was about 1 × 10^−14^ S/m, whereas the electrical conductivity of the sample inside the surface was about 1 × 10^−1^ S/m. Because the major electron transport pathways are mostly internal of the sample, the charge flow in the CNT/EP coating can operate more reliably against surface damage.

### 3.2. Sensing Performance of CNT/EP Coating for Static and Cyclic Temperature

**Figure 5:**
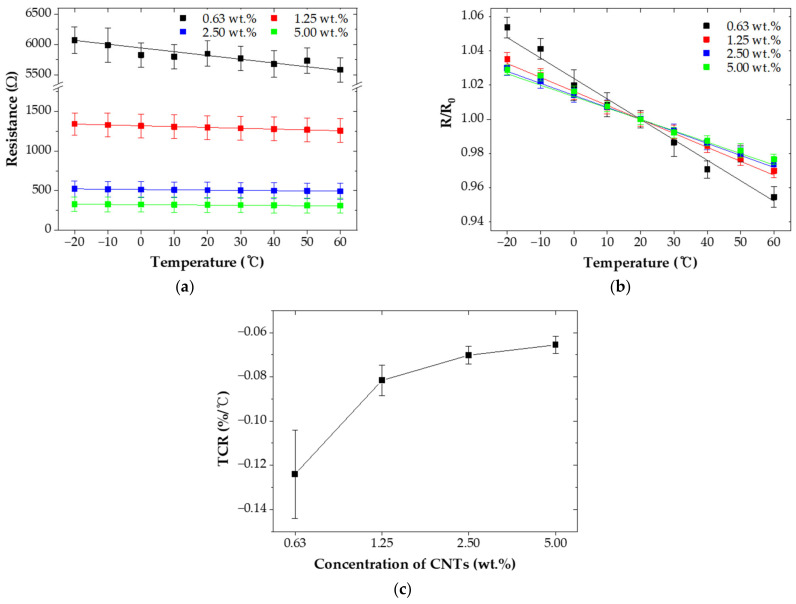
Temperature sensing characteristics of CNT/EP coating; (**a**) electrical resistance; (**b**) normalized resistance; (**c**) TCR.

Figure 5 showed the effect of temperature on the resistance of the CNT/EP coatings. As shown in Figure 5a, the resistance of all CNT/EP coatings gradually decreased with the increasing temperature, demonstrating that CNT/EP coatings have a negative temperature coefficient of resistance (TCR). This phenomenon can be attributed to the thermal activation of charge carriers due to the increase in temperature, which overcomes the potential barrier between CNTs and reduces the resistance of the nanocomposite [26,27,28]. Figure 5b presented the normalized resistance relative to room temperature of the CNT/EP coatings. In this study, the resistance of all CNT/EP coatings varied linearly with temperature except for 0.63 wt.% CNT/EP coating due to its high sensitivity. Figure 5c showed the TCR of CNT/EP coatings. TCR can be calculated by:

(2) $$\mathrm{TCR}=\frac{\left(R\;-{\;R}_{\mathrm{ref}}\right)\;}{R_{\mathrm{ref}}\left(T\;-{\;T}_{\mathrm{ref}}\right)}$$

where, T is the coating temperature in degrees Celsius (°C), T_ref_ is reference temperature (20 °C), R is the resistance at temperature T (Ω), and R_ref_ is resistance at temperature T_ref_ (Ω). It was observed that absolute TCR of CNT/EP coatings decreased with increasing CNT concentrations, and the difference was smaller at high concentrations [17,29]. In this study, TCR values of the CNT/EP coatings are around −0.07%/°C, which is slightly lower than that of the pure carbon sample −0.2%/°C [30,31,32]. Note that the behavior is similar value over the entire measured temperature range. In Table 1, we compared the TCR values obtained from the present study against other temperature sensors. This comparison confirms that the resistances of the CNT/EP coating were measured as functions of temperature [33,34,35,36,37,38].

**Figure 6:**
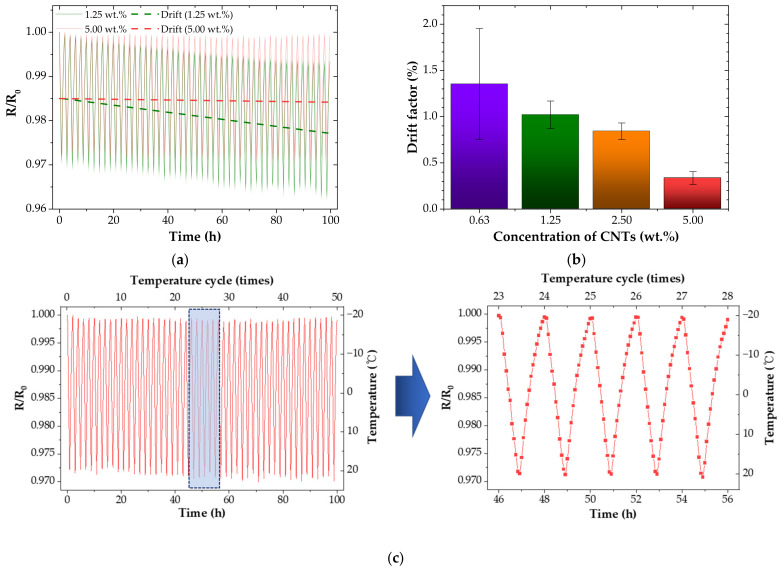
Cyclic temperature sensing performance of the CNT/EP coatings; (**a**) normalized resistance (1.25 wt.%, 5.00 wt.%); (**b**) drift factor; (**c**) sensing performance (5.00 wt.%).

Repeatability is one of the major factors required for long-term stable use of temperature sensors. Figure 6a showed the cyclic piezoresistive response of CNT/EP coatings at different temperatures. It was observed that a drift occurs for both CNT/EP coatings as increasing temperature cycles. A similar phenomenon was reported in previous literatures [39,40]. Some CNTs with weak adhesion may be separated due to the mismatch of the coefficient of thermal expansion during the temperature cycle iteration [41]. Figure 6b presented the drift factor of all CNT/EP coatings. A drift factor can be calculated by

(3) $$\mathrm{Drift}\;\mathrm{factor}=\frac{\left(R_{n}-{\;R}_{0}\right)\;}{R_{0}}\times 100$$

where R_n_ is the resistance of nth sensing cycle (Ω), and R_0_ is the resistance of initial temperature cycle (Ω). Drift significantly reduced with increasing CNT concentrations. For example, the drift factor of the 5.00 wt.% coating is only 0.2% and showed constant repetition. This consistent resistance change demonstrates the long-term and repeatable use of CNT/EP coatings. Figure 6c showed the cyclic piezoresistive response of CNT/EP coating (5.0 wt.%) for 50 temperature cycles. The resistance of the CNT/EP coating was quickly varied with the cyclic temperature change. The proposed coating can clearly monitor the temperature with high stability.

### 3.3. Application of Temperature Sensing System

**Figure 7:**
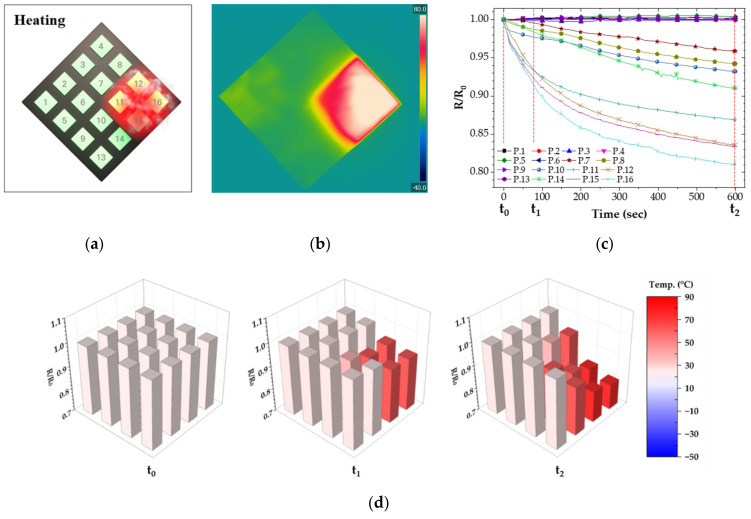
Resistance of CNT/EP coating according to partially heating; (**a**) measured points and applied area; (**b**) infrared thermal image; (**c**) normalized resistance; (**d**) normalized resistance at t_0_, t_1_, t_2_.

**Figure 8:**
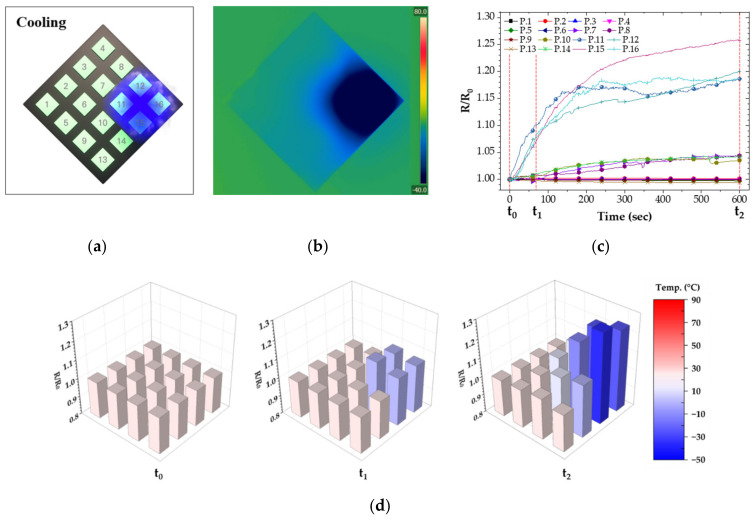
Resistance of CNT/EP coating according to partially cooling; (**a**) measured points and applied area; (**b**) infrared thermal image; (**c**) normalized resistance; (**d**) normalized resistance at t_0_, t_1_, t_2_.

To demonstrate the feasibility of the temperature sensor, we placed CNT/EP coating on polyethylene terephthalate (PET) substrate to install a temperature sensor array for simulating temperature distribution. The heat source was applied to a corner of CNT/EP coatings to show heat distribution for the sensor array, as shown in Figure 7a and Figure 8a. Also, an infrared thermal camera was used to monitor heat distribution for CNT/EP coating (Figure 7b and Figure 8b). Before applying the heat source, each sensor maintained its initial resistance. After the heat source applies to a corner of CNT/EP coating, the resistance of sensors near the heat source dramatically changes with time, as shown in Figure 7c and Figure 8c. As a result of measuring resistance according to heat source, the resistance near the heat source part was significantly increased or reduced with time. The normalized resistance near the heat source part changed with the temperature, but there is little change in the other part. In particular, the resistance of sensors prominently changed in the heat source area, as shown in Figure 7d and Figure 8d. Temperature sensing can be possible through the resistance according to partial temperature change of the coating, which means that the CNT/EP coating can function as a temperature sensor and respond to various fields.

## 4. Conclusions

This study investigated the characteristics of CNT/EP coatings as a temperature sensor. Highly electrically conductive coatings have been successfully prepared by dispersing CNTs in an epoxy matrix. The addition of CNTs showed improved electrical conductivity. Static temperature sensing experiments confirmed that the proposed coating provided temperature sensing due to its negative temperature coefficient and can clearly monitor the temperature with high stability. Through the cyclic temperature experiment, it was confirmed that the drift of the CNT/EP coating occurred as the number of repetitions increased, which was alleviated as the CNT concentration increased. As a result, CNT/EP coatings with higher CNT concentrations are more suitable for temperature monitoring. In addition, the potential as a temperature sensor was confirmed through the partial temperature change experiment of the CNT/EP coating. Therefore, CNT/EP coating may enable potential applications to temperature sensors and sensor-integrated materials.

## Figures and Tables

**Table 1:** Comparison of the TCR between our case and other types of temperature sensors.

Reference	Material	|TCR| (10^−3^/°C)	Measured Temperature Range (°C)
In this paper (Maximum)	CNT/epoxy	0.7	−20 to 60
[33]	CNT-GO	~60	5–50, 25–70
[34]	CNT/PDMS; CNT/FG/PDMS;	1.5–2.8 ~28	40–80
[35]	MWCNT	2.4–2.7	20–75
[36]	CNT yarn (without solvent)	~0.75	25–80
[37]	SWNT-CMC	~3	0–100
[38]	CNT deposited on ITO electrodes	~0.4	25–90

## Data Availability

Not applicable.
